# Telomere length correlates with physiological and behavioural responses of a long-lived seabird to an ecologically relevant challenge

**DOI:** 10.1098/rspb.2022.0139

**Published:** 2022-07-13

**Authors:** Z. M. Benowitz-Fredericks, L. M. Lacey, S. Whelan, A. P. Will, S. A. Hatch, A. S. Kitaysky

**Affiliations:** ^1^ Department of Biology, Bucknell University, Lewisburg, PA, USA; ^2^ Department of Natural Resources Sciences, McGill University, Ste-Anne-de-Bellevue, Quebec, Canada; ^3^ Institute of Arctic Biology, Department of Biology and Wildlife, University of Alaska Fairbanks, Fairbanks, AK, USA; ^4^ Bioscience Group, National Institute of Polar Research Japan, 10-3, Midori-cho, Tachikawa, Tokyo 190-8518, Japan; ^5^ Institute for Seabird Research and Conservation, Anchorage, AK, USA

**Keywords:** biologging, corticosterone, food availability, glucocorticoids, life history, telomeres

## Abstract

Determinants of individual variation in reallocation of limited resources towards self-maintenance versus reproduction are not well known. We tested the hypothesis that individual heterogeneity in long-term ‘somatic state’ (i) explains variation in endocrine and behavioural responses to environmental challenges, and (ii) is associated with variation in strategies for allocating to self-maintenance versus reproduction. We used relative telomere length as an indicator of somatic state and experimentally generated an abrupt short-term reduction of food availability (withdrawal of food supplementation) for free-living seabirds (black-legged kittiwakes, *Rissa tridactyla*). Incubating male kittiwakes responded to withdrawal by increasing circulating corticosterone and losing more weight compared to continuously supplemented controls. Males with longer telomeres increased time in directed travel regardless of treatment, while experiencing smaller increases in corticosterone. Males with longer telomeres fledged more chicks in the control group and tended to be more likely to return regardless of treatment. This study supports the hypothesis that somatic state can explain variation in short-term physiological and behavioural responses to challenges, and longer-term consequences for fitness. Male kittiwakes with longer telomeres appear to have prioritized investment in self over investment in offspring under challenging conditions.

## 1. Background

The life of free-living animals is rife with challenges that require them to continuously adjust their physiology and behaviour in ways that maximize their fitness. Fluctuations in resource availability are an important environmental challenge that forces reallocation of energy between processes of somatic maintenance and reproduction. Individuals' responses to fluctuations in resource availability vary along the life-history strategy continuum, even within a population (e.g. [1]). Individuals are expected to adopt different allocation strategies, and this variation may manifest most strongly under challenging conditions [2]. An individual's strategy presumably reflects an integration of its internal resources and constraints with current environmental conditions in ways that maximize fitness [3]. While a multitude of genetic and environmental factors contribute to variation in strategies, much research has focused on glucocorticoids as a physiological mediator of resource allocation [4], and telomeres as an integrative indicator of long-term ‘somatic state’ that may correlate with allocation decisions [5].

### (a) Glucocorticoids and energy allocation

In most vertebrates, a primary mediator of physiological and behavioural reallocation in response to challenges is a transient increase in circulating glucocorticoids, regulated by the hypothalamic–pituitary–adrenal (HPA) endocrine axis. Seabirds are long-lived top predators with life-histories that are primarily regulated by bottom-up processes—their glucocorticoid (corticosterone) levels often vary in response to the availability and quality of their prey at sea [6,7] and elevated corticosterone is often interpreted as an indicator of nutritional stress [8–11]. At the scale of colonies, elevated baseline corticosterone can be associated with reduced reproductive success [12,13].

Black-legged kittiwakes (*Rissa tridactyla*)—small, long-lived, colonial, cliff-nesting gulls—have been the focus of many experiments examining the interactions among food availability, corticosterone and behaviour. Though responses can vary based on factors like sex and body condition, kittiwakes tend to respond to exogenous corticosterone by spending less time attending the nest and more time foraging at sea [14–16]. Corticosterone-implanted kittiwakes may subsequently suffer reduced survivorship [15,17] and other costs, like faster declines in telomere length [18]. However, as in many species, populations of kittiwake show substantial heterogeneity in the relationship between corticosterone and life-history strategy. In one study, a Pacific kittiwake population responded to exogenous corticosterone by decreasing nest attendance, such that chicks bore the costs of elevated parental corticosterone (reflected in reduced growth and survival), while Atlantic kittiwakes showed the opposite response, buffering chicks and incurring direct costs [13]. Further, there is heterogeneity in both the magnitude and consequences of glucocorticoid elevation in response to challenges within populations [19]. Efforts to identify factors contributing to this variation have converged on telomere length as an indicator of somatic state with potential to explain interindividual variation in behavioural [20] and physiological responses to challenges, as well as variation in life-history strategies [21].

### (b) Telomeres, somatic state and life history

Telomeres are vital, non-coding DNA ‘caps’ at the ends of chromosomes. Telomere length varies at birth, is affected by both genetic and epigenetic factors, and is thought to be regulated by cellular processes such as cell division, metabolic rate and, potentially, oxidative damage (reviewed in [22]) that may reduce telomere length. In some species, the ongoing activity of the enzyme telomerase increases telomere length [23]. While there are multiple hypotheses about sources of interindividual variation in telomere length, telomeres are frequently used as a proxy for long-term somatic state, a measure of physiological deterioration that is the product of genetics and developmental experience, exposure to physiological challenges and allocation to investment in somatic maintenance and repair [24]. While chronological age can explain some variation in telomere length [25], relative telomere length (RTL) may be better explained by the cumulative consequences of an individual's life-history strategy and exposure to environmental challenges; there is evidence that individuals allocating more intensively to reproduction incur costs to their own self-maintenance that manifest in shorter telomeres (the ‘Biological Age’ or ‘Reproductive Cost’ hypothesis [26–29]). However, telomere length has also been proposed as a potential proxy for individual quality in some species. Some seabird studies found no relationship between chronological age and telomere length, and no evidence that increased reproductive effort is associated with shorter telomeres [1]. These correlative studies instead support the ‘Telomere-Individual Quality’ hypothesis, where individuals with longer telomeres are better able to navigate environmental challenges and have higher reproductive success [30,31]. More recently, the ‘Telomere Messenger’ hypothesis proposes that telomere dynamics are an adaptive mechanistic mediator of individual life-history strategies, acting as a signal that determines how an individual alters energy allocation in response to environmental conditions to maximize their fitness [32]. Whether telomere length in kittiwakes reflects reproductive costs, individual quality or both [33], it is a strong candidate for explaining interindividual variation in relative allocation to self-maintenance and reproduction in response to ecological challenges. Though relationships between corticosterone exposure and telomere length vary, corticosterone has been proposed as a link between telomere length and other aspects of phenotype [21,26,34,35].

### (c) Ecologically relevant challenge

In this study, we experimentally reduced food availability for free-living adult kittiwakes of known age to test the ability of telomere length to explain interindividual variation in responsiveness to an ecologically relevant challenge. We capitalized on the unique ability to supplementally feed free-living adult kittiwakes at a field site on Middleton Island, AK. By withdrawing food supplementation from incubating males who had been continuously provided with fish at the nest ad libitum since the start of the year's breeding season, we generated an ecologically relevant challenge that we anticipated would elevate baseline plasma corticosterone, in response to (i) a change in energy balance due either to decreased food intake or increased effort required to acquire food, and/or (ii) the perception that food availability was unpredictable [36,37]. We measured baseline corticosterone, body mass and GPS-derived movement behaviour before and after the experimental withdrawal of supplemental food. We also measured reproductive success in the year of the experiment and noted the birds' return to the colony in the subsequent year. We used this experimental manipulation to test the hypothesis that heterogeneity in individuals’ somatic state explains variation in (i) endocrine and behavioural responses to short-term environmental challenges, and (ii) relative investment in current versus future reproduction. The hypothesis predicts that telomere length, as an indicator of somatic state, will correlate with physiological and behavioural responses to this ecologically relevant challenge, as well as variation in individual strategies for allocating to self-maintenance versus a given reproductive event.

## 2. Methods

### (a) Study site

Black-legged kittiwakes nest on a modified radar tower on Middleton Island, AK (59.4283° N, 146.3300° W). The tower and food supplementation regimen have been described in detail previously [38], but briefly: kittiwakes voluntarily nest on horizontal wooden beams on the outside walls of the tower; wood partitions delineate nest sites. Individual nest sites are visible and accessible from inside the tower via sliding mirrored glass windows. Holes with PVC tubes allow delivery of whole thawed capelin (*Mallotus villosus*) to each nest. All birds are sexed based on behaviours during courtship and copulation and are banded with unique colour combinations. Nests are monitored daily to determine lay, hatch and fledge dates.

### (b) Experimental design

**Figure 1.**
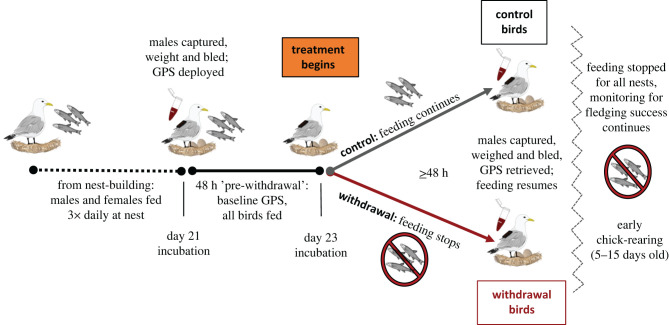
Experimental timeline for sampling and food withdrawal. (Online version in colour.)

In May 2017, we began training birds to accept supplemental food at 20 nests on a single panel of the tower (figure 1). The panel had not been supplemented for the four consecutive years prior to this study (and only one bird from 2017 was fed in 2013). Feeding began on 8 May, after pairs had been established at nest sites. Nest occupants were fed as many capelin as they would consume three times per day (at 8.00, 14.00 and 18.00 h).

The male at each nest containing at least one egg was captured 21 days after its first egg was laid (incubation is approx. 28 days), beginning on 18 June. We focused on male kittiwakes because they are more likely than females to maintain investment in self-maintenance than reproduction [39] and were thus expected to be more behaviourally responsive to food withdrawal than females. We conducted the study during incubation because while incubation is energetically costly [40], birds may be more responsive to environmental challenges than during chick rearing.

Birds were captured from inside the tower and bled from the alar vein within 3 min after initial disturbance to acquire baseline corticosterone levels [41]. Birds were then weighed and fitted with a GPS unit (i-gotU GT-120 sealed in small heat-shrink pouches; 16.7 ± 0.1 g; 2 min fix rate), affixed to two central rectrices at the base of the tail below the preen gland using marine cloth tape (Tesa), glue and cable ties.

Birds carrying GPS units were released and food supplementation continued for approximately 48 h (the duration of six scheduled feeds) for all nests, to acquire baseline (pre-withdrawal) measures of movement behaviour, after which 11 nests stopped receiving food supplementation (withdrawal treatment) while nine nests continued to receive food supplementation (control treatment). Because kittiwakes retained the ability to forage freely at sea, withdrawal of food supplementation increased the effort required to acquire food without preventing access to food [42]. The 2017 season followed a multi-year marine heat wave and was a relatively low-food year for kittiwakes at this site, with incubating birds travelling long distances [43]. Thus, birds in this experiment may have been particularly reliant on food supplementation, potentially magnifying the impacts of withdrawal of the supplement.

Treatments were assigned in alternation by lay date. Target capture time was 48 h after food withdrawal (96 h after initial capture), but one bird was captured 2 days late, and a second 4 days late. At recapture, birds were immediately bled again, relieved of their GPS units, re-weighed, and released. Food supplementation was resumed for at least a week after recapture then withdrawn from all birds for the remainder of the breeding season owing to logistical constraints (chick ages: 5–15 days).

### (c) Reproductive success and return to colony

All nests were monitored daily until the end of the breeding season, and nests were categorized as successfully fledging a chick if a chick either left the nest after 35 days or was at least 35 days old and still in the nest when monitoring ended for the season (mortality at the colony after this age is rarely observed).

Daily resighting the following summer identified every individual who returned to nest at a tower nesting site, as well as hundreds of individuals nesting at other locations on the island. Because nest site fidelity and resighting probability are high in this population, birds that were not resighted in 2018 were interpreted as not having returned to the colony to breed.

### (d) Hormone analyses

Hormone analysis was conducted blind to treatment. Whole blood was kept cool for up to 1 h after collection in the field, then centrifuged (5 min at 7500 r.p.m.) to separate plasma and red blood cells, which were kept frozen until assay. Corticosterone was assayed in duplicate in a single radioimmunoassay, as previously described [6]. Average recovery of radiolabelled corticosterone after extraction was 94%, and the coefficient of variation (CV) of duplicates was 1.6%. Corticosterone samples were not available for three birds.

### (e) Telomere analyses

Telomere analysis was conducted blind to treatment. Blood samples from 10-day old kittiwake chicks from fed nests in 2016 were also included in the analysis (samples collected and handled as for adults). DNA was extracted from frozen red blood cells from pre-withdrawal blood samples (stored temporarily at −20°C in the field, then at −80°C until analysis), and telomere length quantified using real-time quantitative polymerase chain reaction (qPCR). Briefly, we extracted DNA using DNeasy Blood and Tissue Kit (Qiagen), assessed extract concentration and purity using a NanoDrop spectrophotometer, and stored in elution buffer at −80°C. DNA concentration of all samples was at least 20 ng µl^−1^ and of high quality (260/280 ratio averaged 1.91 ± 0.101 (s.d.), and 260/230 ratio averaged 2.11 ± 0.102). To confirm behaviour-based sexing, we genetically sexed all birds following Young *et al*. [44].

We measured RTL using real-time qPCR. This technique provides telomere quantity as a ratio of telomeric DNA to the quantity of a single-copy reference gene (here GAPDH). General protocols were adapted from Cawthon [45], reference gene primers were from Criscuolo *et al.* [46] and telomere primers were from Foote [47]. Samples were run in triplicate, on different plates for the telomere and the reference gene primers. The qPCR protocol for the reference gene (GAPDH) was: 2 min at 50°C, then 10 min at 95°C, followed by 40 cycles of 15 s at 95°C and 1 min at 60°C. For the telomere reaction it was 2 min at 50°C, then 10 min at 95°C, followed by 40 cycles of 15 s at 95°C, 20 s at 58°C and 1 min at 72°C. Mean intra-plate CV's (calculated as s.d. among three replicates divided by the average Cq value) were 0.72% for GAPDH and 0.94% for telomeres. Efficiencies of amplification (calculated from a standard curve included on each plate) were 100/101% and 90/97.5% for GAPDH and for telomeres in chick and adult assays, respectively. Repeatability of the RTL measurements, determined using an intra-class correlation coefficient (ICC) [48], was high (ICC = 0.95). An identical standard sample was run on each plate. Since efficiencies differed, we used Cawthon's [45] method of RTL calculation, which corrects for the primer efficiency and standardizes to the standard sample so that values can be compared across plates.

#### (i) Movement analyses

All movement analyses were carried out using the *moveHMM* [49] package in R (v. 3.4.1). We analysed tracks of 18 males; one withdrawal male lost the device during deployment and a second withdrawal male was excluded because the device recorded data for only part of the experiment. Parameters for the model were first maximized using a recursive algorithm and selected based on the resulting models estimated maximum-likelihood ratio [50]. With the resultant parameters, we used a hidden Markov model with a wrapped Cauchy angle distribution to identify bird movement as one of two states based on step length and turn angle [50]. State 1 was characterized by long step lengths and low turn angles and can be described as ‘directed flight’; it is usually associated with transit flight to or from the colony. State 2 is characterized by short step lengths and high turn angles—it was highly correlated with time spent at or very near the colony, though we cannot differentiate between loafing near the nest, and ‘foraging’ at the nest (waiting for supplemental feeding). The amount of time spent in each state was converted into the proportion of total time in the pre- and post-withdrawal windows, and the change in proportion of time spent in state 1 was used as an indicator of behavioural change.

### (f) Statistical analyses

To account for individual variation in behaviour and physiology, the independent variable used for analyses (for body mass, corticosterone and movement behaviour) was the change from pre- to post-. See the electronic supplementary material, table S1 for sample sizes, initial and final values for all measures by treatments; and S3 for full model results. All statistical analyses were run in R (v. 3.6.2; see the electronic supplementary material, S4 for code). To first determine whether the food withdrawal treatment posed a physiological challenge, we compared the magnitude of change in corticosterone and the change in body mass (from pre-withdrawal capture to post-withdrawal capture) between control and withdrawal treatments using Welch's two-sample *t*-tests (one-tailed). We then fitted linear models and generalized linear models (GLM) and log-transformed non-normal response variables to achieve normality. We used Type III ANOVA to test significance of interactions; when interactions were non-significant, we removed the interaction term and used Type II ANOVA to test for main effects. We report model estimates ± s.e.

#### (i) Age and telomere length

To test whether telomere length reflects chronological age, we modelled RTL in response to age. We first used data from the subset of known-age adults that were banded as chicks at the colony (*n* = 12). Then, we repeated the analysis including conservative age estimates for birds that were not banded as chicks (*n* = 8), which were calculated as year first banded at the colony plus 7 years, the average age of recruitment for birds at historically unfed nests at this colony [51]. Finally, we modelled log-transformed RTL including all experimental adults and also 10-day old chicks.

#### (ii) Telomere length and variation in response to challenge

To test the hypothesis that heterogeneity in individuals' somatic state explains variation in endocrine and behavioural responses to short-term environmental challenges, we modelled change in corticosterone and change in the proportion of time spent in movement state 1, in response to treatment, RTL, and their interaction. To assess whether somatic state explained physiology and behaviour in the absence of an environmental challenge, we modelled pre-withdrawal corticosterone (at first capture; log-transformed) and pre-withdrawal proportion of time spent in movement state 1 (first 48 h; log-transformed) in response to RTL.

#### (iii) Telomere length and life history

To test the hypothesis that heterogeneity in individuals' somatic state is associated with different strategies for optimizing fitness, we modelled the number of chicks fledged in response to treatment, RTL and their interaction. We used logistic regression to model return to colony the following year (yes/no; binomial GLM) in response to telomere length, treatment and their interaction. Where interactions were significant, separate *post hoc* models were re-run for each treatment in response to RTL only.

## 3. Results

### (a) Response to food withdrawal

**Figure 2.**
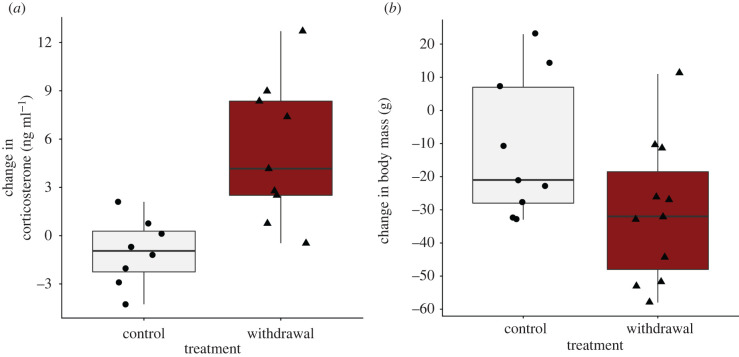
Male kittiwakes subject to greater than 48 h of experimental withdrawal of supplemental feeding at the nest had (*a*) higher increases in baseline corticosterone and (*b*) larger decreases in body mass than control birds with uninterrupted supplemental feeding. (Online version in colour.)

The increase in corticosterone was significantly greater in withdrawal birds than controls (figure 2*a*; *t*_11.7_ = −3.9, *p* < 0.01). Withdrawal birds also lost more body mass between the two capture events (figure 2*b*; *t*_17_= 2.0, *p* < 0.05).

### (b) Age and telomere length

Among adult males included in this experiment, there was no relationship between telomere length and age using either birds with known ages (*n* = 12, range 7–17 years; *β* = 0.029 ± 0.064, *F*_1,10_ = 0.20, *p* = 0.66) or combined known age and estimated age birds (*n* = 19, range 7–18 years; *β* = 0.028 ± 0.044, *F*_1,18_ = 0.39, *p* = 0.54). However, telomere length declined with age when we included both experimental adults and 10-day old chicks (electronic supplementary material, figure S1; *β* = −0.024 ± 0.006, *F*_1,52_ = 16.1, *p* < 0.001).

### (c) Telomere length and variation in response to challenge

#### (i) Corticosterone

Telomere length did not explain any variation in (log-transformed) baseline corticosterone levels at first capture (*β* = 0.35 ± 0.86, *F*_1,17_ = 0.17; *p* = 0.69).

**Figure 3.**
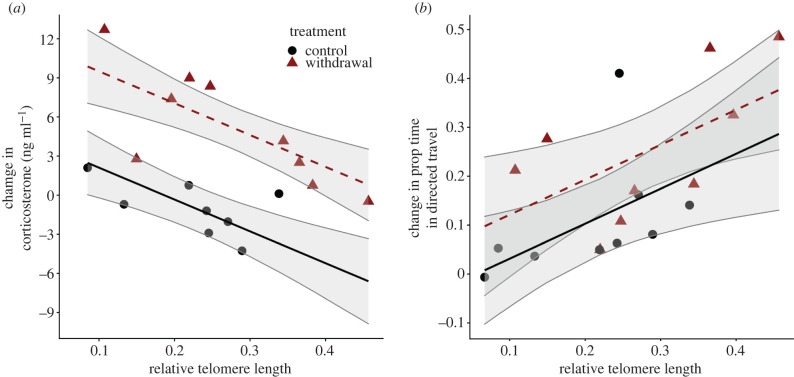
Telomere length explained individual heterogeneity in (*a*) changes in baseline corticosterone and (*b*) changes in movement behaviour (proportion of time spent in directed travel). Lines indicate model predictions with 95% confidence intervals. (Online version in colour.)

Telomere length and treatment did not interactively influence the change in baseline corticosterone from pre- to post-withdrawal sampling (*β* = −15.6 ± 13.4, *F*_1,13_ = 1.4, *p* = 0.26). However, withdrawal birds increased baseline corticosterone more than controls (*β* = 7.4 ± 1.2, *F*_1,14_ = 35.7, *p* < 0.0001; figure 3*a*) and individuals with shorter telomeres increased baseline corticosterone more than individuals with longer telomeres (*β* = −24.4 ± 6.2, *F*_1,14_ = 15.5, *p* < 0.01; figure 3*a*).

#### (ii) Movement behaviour

Telomere length was not associated with variation in the (log-transformed) proportion of time in state 1 in the first 48 h. (i.e. prior to food withdrawal; *β* = 1.0 ± 0.59, *F*_1,16_ = 0.45, *p* = 0.51), however the change in proportion of time spent in directed travel (state 1) was significantly explained by telomere length (*β* = 0.72 ± 0.30, *F*_1,15_ = 6.5, *p* < 0.05)—birds with longer telomeres had a larger increase in time spent in directed travel between pre- and post-withdrawal time periods (figure 3*b*). Treatment effect was not statistically significant (treatment × telomere length: *β* = 0.18 ± 0.59, *F*_1,14_ = 0.10, *p* = 0.76; treatment: *β* = 0.090 ± 0.059, *F*_1,15_ = 2.3, *p* = 0.15).

### (d) Telomere length and life history

#### (i) Reproductive success

**Figure 4.**
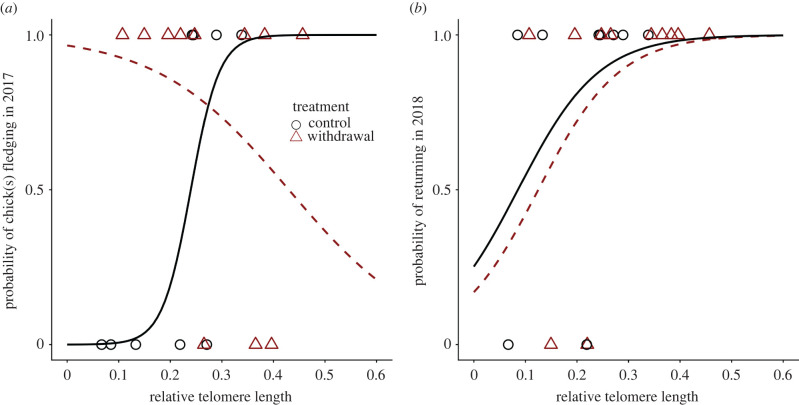
(*a*) Male kittiwakes with continuous access to supplemental food (controls) were more likely to fledge a chick if they had longer telomeres; the relationship between telomere length and reproductive success in the withdrawal group was not statistically significant. (*b*) Individuals with longer telomeres tended to be more likely to be resighted at the colony the following year, regardless of treatment. Lines indicate model predictions. (Online version in colour.)

An interactive effect between treatment and telomere length indicated that the association between telomere length and fledging success varied between treatments (treatment × telomere length: *β* = −43.9 ± 30.4, *χ*^2^_1_ = 6.9, *p* < 0.01; treatment: *β* = 12.0 ± 7.8, *χ*^2^_1_ = 8.0, *p* < 0.01; telomere length: *β* = 36.1 ± 29.5, *χ*^2^_1_ = 5.9, *p* < 0.05; figure 4*a*). *Post hoc* analyses showed a positive relationship between telomere length and fledging success within the control group (*β* = 36.1 ± 29.5, *χ*^2^_1_ = 5.9, *p* < 0.05); though the relationship appears negative in the withdrawal group (figure 4*a*), it was not statistically significant (*β* = −7.8 ± 7.5, *χ*^2^_1_ = 1.3, d.f. = 1, *p* = 0.26).

#### (ii) Return to colony

The probability that birds returned in the following year was not influenced by an interaction between telomere length and treatment (*β* = 2.4 ± 15.1, *χ*^2^_1_ = 0.026, *p* = 0.87). However, birds with longer telomeres were more likely to return to the colony (*β* = 12.7 ± 7.5, *χ*^2^_1_ = 3.8, *p* < 0.05) regardless of treatment (*β* = −0.50 ± 1.30, *χ*^2^_1_ = 0.15, *p* = 0.70; four birds were not resighted in 2018, two from the control group and two from the withdrawal group; all four had RTLs below the 50th percentile; figure 4*b*). Birds that did not return in 2018 were not resighted in the subsequent 4 years.

## 4. Discussion

We found that male kittiwakes with longer telomeres had smaller increases in corticosterone, larger adjustments in movement behaviour, and were more likely to invest in self-maintenance in the face of an abrupt reduction in local food availability. Of note, RTL did not explain baseline corticosterone at initial capture, or behaviour in the first 48 h prior to the withdrawal of food, suggesting that relationships between somatic state and individual heterogeneity may be more likely to be revealed under challenging conditions. In other words, our experimental manipulation of resource availability allowed underlying variation in resource allocation strategies to emerge [52]. In this study, we were able to control for many potential sources of variation—sex, age, and reproductive stage—by using known-age male kittiwakes at day 21 of incubation and measuring baseline corticosterone and behaviour before and after experimental manipulation.

### (a) An ecologically relevant challenge

Kittiwakes exhibit fidelity to local foraging patches [53], yet the existence of such patches can be ephemeral, necessitating adjustments in foraging behaviour [54]. We mimicked the disappearance of a local food patch by removing the daily food supplement that had been a reliable source of food for approximately five weeks. The removal of this food supplement for 48 h more than doubled baseline corticosterone for free-living male kittiwakes with unhindered access to foraging at sea, though levels remained well below reported capture-and-handling induced maxima for kittiwakes at this site (approx. 35–45 ng ml^−1^ [9]). This reduction in local food availability also revealed a relationship between telomere length and baseline corticosterone levels that was previously masked. Most studies that have used experimental manipulations to explore relationships between corticosterone and RTL in free-living animals have relied on the administration of exogenous corticosterone. The physiological consequences of exogenous corticosterone administration are complicated, with potentially undesirable consequences such as inhibition of the HPA axis following an initial spike [55,56]. In fact, administration of exogenous corticosterone has also been used as a tool to shut down endogenous glucocorticoid production in seabirds [57]. By experimentally inducing variation in endogenous glucocorticoid secretion rather than relying on exogenous administration, we were able to explore the concurrent changes in movement behaviour and reveal a correlation between telomere length and heterogeneity in these responses. However, causal relationships among telomere length, corticosterone secretion and behaviour are difficult to disentangle.

### (b) Telomeres, corticosterone and behaviour

Our study cannot differentiate between the possibility that HPA axis reactivity drives RTL (by generating variation in exposure to telomere-shortening glucocorticoids (e.g. [34])) and the possibility that somatic state/telomere length drives HPA axis reactivity, perhaps acting as a ‘messenger’ that integrates past experience and optimizes phenotype accordingly [32]. In many long-lived seabirds including kittiwakes (electronic supplementary material, figure S1), a substantial amount of the variation in adult telomere length seems to be driven by telomere attrition during development [58]. Factors during the nestling phase such as social milieu (i.e. brood size/degree of competition [44]), interannual variation (presumably in resource availability [59,60]) or exposure to elevated glucocorticoids [28] can generate variation in telomere length. However, while challenging experiences during adulthood such as increased energy expenditure and elevated glucocorticoid exposure [18] can accelerate telomere attrition in some species (potential mechanisms reviewed in [34,35]), a recent meta-analysis suggests that short-term elevations in glucocorticoids, at least, do not consistently alter telomere length [61].

Relatively few studies have looked for relationships between movement behaviour and telomere length. In our study, RTL explained some of the change in movement behaviour, with longer telomere birds showing a larger increase in directed travel, regardless of treatment group. Potential explanations for the increase in proportion of time in state 1 in the control group (all values positive, no treatment effect) include a change in environmental conditions, sensitivity of control birds to the disrupted food availability at neighbouring nests, or the possibility that GPS deployment (relatively heavy at approx. 17 g or 3.5% body mass) may have served as an additional challenge that triggered differential behavioural responses [62] associated with telomere length. The two general categories of explanations for relationships between telomere length and behaviour are that (i) telomere length drives the adoption of particular behavioural strategies (selective adoption), or (ii) behaviour is causal and the cumulative consequences of foraging strategies have contributed to variation in telomere length [63]. In several bird species, individuals with what appear to be more efficient foraging strategies have longer telomeres or experience less telomere loss [30,44,64]. In this study, male kittiwakes with longer telomeres appeared more behaviourally responsive to challenges, despite showing smaller increases in corticosterone. The increase in the proportion of time in directed flight by longer telomere birds may reflect an environment-driven shift in foraging strategy from ‘sit and wait at a local patch’ (food supplementation at the nest) to ‘travel to seek food elsewhere’, perhaps at distant but predictable food patches (*sensu* [53]) that become worthwhile under more challenging conditions. While we would predict that other populations or species would also show a relationship between telomere length and changes in foraging strategies in response to environmental challenges, what constitutes an optimal response strategy is likely to vary depending on the distribution and predictability of food.

### (c) Telomeres and life-history

Longer telomere birds tended to have higher return rates the following year, but only had higher reproductive success in the absence of the short-term food withdrawal challenge (control group). The second food withdrawal, when we stopped supplementing all nests in early chick rearing, probably served as an additional energetic challenge, such that variation in reproductive success and return rates reflected responses to either one (control group), or two (withdrawal group) challenges. Our results are consistent with studies showing that individuals with longer telomeres tend to adopt a ‘slower’ pace of life strategy, keeping corticosterone low and prioritizing investment in self-maintenance over a single reproductive event when conditions become challenging. This is simultaneously consistent with longer telomere birds maintaining better somatic state, and with past experimental evidence that unlike their Atlantic conspecifics, kittiwakes at this Pacific colony allocate towards self-maintenance over reproduction in the face of elevated corticosterone [13]. We would predict that, in contrast to their Pacific counterparts, Atlantic kittiwakes with longer telomeres would show higher reproductive success and, potentially, lower survival in the face of a decline in resource availability.

## 5. Conclusion

This study supports the hypothesis that, in this long-lived seabird species, somatic state may be able to explain variation in both short-term physiological and behavioural responses to challenges, and the longer-term consequences for fitness. Though studies in additional years, populations and species are needed before these results can be generalized, our results suggest that in our experimental subset of incubating male Pacific kittiwakes, birds with longer telomeres appear to be ‘prudent parents’ who, under challenging conditions, responded physiologically and behaviourally in ways that prioritized maintenance of self (somatic state) over investment in offspring [65]. The ability of telomere length to explain variation in response to an environmental challenge is predicted by most hypotheses about the relationships among telomeres, somatic state and resource allocation but is perhaps surprising given our relatively small sample size. We hypothesize that our non-pharmacological experimental manipulation, combined with our ability to control for multiple potential sources of variation and to measure individual responses (rather than just initial or final states), revealed these relationships. We suggest that future studies exploring the relationship between telomere length and heterogeneity in phenotype/fitness may benefit from using ecologically relevant challenges to elicit underlying heterogeneity in responses, instead of relying on variation at a single time point.

## Data Availability

Data is available from the Dryad Digital Repository: https://doi.org/10.5061/dryad.v9s4mw6xs [66]. R code used for analyses is in the electronic supplementary material. Electronic supplementary material is available online [67].

## References

[1] Young RC, Kitaysky AS, Drummond HM. 2021 Telomere lengths correlate with fitness but assortative mating by telomeres confers no benefit to fledgling recruitment. Sci. Rep. **11**, 5463. (10.1038/s41598-021-85068-x) 33750872 PMC7943796

[2] Vedder O, Bouwhuis S. 2018 Heterogeneity in individual quality in birds: overall patterns and insights from a study on common terns. Oikos **127**, 719-727. (10.1111/oik.04273)

[3] Stearns SC. 2000 Life history evolution: successes, limitations, and prospects. Naturwissenschaften **87**, 476-486. (10.1007/s001140050763) 11151666

[4] Crespi EJ, Williams TD, Jessop TS, Delehanty B. 2013 Life history and the ecology of stress: how do glucocorticoid hormones influence life-history variation in animals? Funct. Ecol. **27**, 93-106. (10.1111/1365-2435.12009)

[5] Young AJ. 2018 The role of telomeres in the mechanisms and evolution of life-history trade-offs and ageing. Phil. Trans. R. Soc. B **373**, 20160452. (10.1098/rstb.2016.0452) 29335379 PMC5784072

[6] Kitaysky A, Piatt J, Wingfield J. 2007 Stress hormones link food availability and population processes in seabirds. Mar. Ecol. Prog. Ser. **352**, 245-258. (10.3354/meps07074)

[7] Will A et al. 2020 The breeding seabird community reveals that recent sea ice loss in the Pacific Arctic does not benefit piscivores and is detrimental to planktivores. Deep Sea Res. Part II Top. Stud. Oceanogr. **181–182**, 104902. (10.1016/j.dsr2.2020.104902)

[8] Benowitz-Fredericks ZM, Shultz MT, Kitaysky AS. 2008 Stress hormones suggest opposite trends of food availability for planktivorous and piscivorous seabirds in 2 years. Deep Sea Res. Part II Top. Stud. Oceanogr. **55**, 1868-1876. (10.1016/j.dsr2.2008.04.007)

[9] Kitaysky AS, Piatt JF, Hatch SA, Kitaiskaia EV, Benowitz-Fredericks ZM, Shultz MT, Wingfield JC. 2010 Food availability and population processes: severity of nutritional stress during reproduction predicts survival of long-lived seabirds. Funct. Ecol. **24**, 625-637. (10.1111/j.1365-2435.2009.01679.x)

[10] Schultner J, Kitaysky AS, Welcker J, Hatch S. 2013 Fat or lean: adjustment of endogenous energy stores to predictable and unpredictable changes in allostatic load. Funct. Ecol. **27**, 45-55. (10.1111/j.1365-2435.2012.02058.x)

[11] Sorenson GH, Dey CJ, Madliger CL, Love OP. 2017 Effectiveness of baseline corticosterone as a monitoring tool for fitness: a meta-analysis in seabirds. Oecologia **183**, 353-365. (10.1007/s00442-016-3774-3) 27873067

[12] Buck CL, O'Reilly KM, Kildaw SD. 2007 Interannual variability of black-legged kittiwake productivity is reflected in baseline plasma corticosterone. Gen. Comp. Endocrinol. **150**, 430-436. (10.1016/j.ygcen.2006.10.011) 17161400

[13] Schultner J, Kitaysky AS, Gabrielsen GW, Hatch SA, Bech C. 2013 Differential reproductive responses to stress reveal the role of life-history strategies within a species. Proc. R. Soc. B **280**, 20132090. (10.1098/rspb.2013.2090) PMC3790493 24089339

[14] Angelier F, Clementchastel C, Gabrielsen G, Chastel O. 2007 Corticosterone and time–activity budget: an experiment with black-legged kittiwakes. Horm. Behav. **52**, 482-491. (10.1016/j.yhbeh.2007.07.003) 17707380

[15] Kitaysky AS. 2001 Corticosterone facilitates begging and affects resource allocation in the black-legged kittiwake. Behav. Ecol. **12**, 619-625. (10.1093/beheco/12.5.619)

[16] Nelson BF, Daunt F, Monaghan P, Wanless S, Butler A, Heidinger BJ, Newell M, Dawson A. 2015 Protracted treatment with corticosterone reduces breeding success in a long-lived bird. Gen. Comp. Endocrinol. **210**, 38-45. (10.1016/j.ygcen.2014.10.003) 25449182

[17] Goutte A, Angelier F, Welcker J, Moe B, Clément-Chastel C, Gabrielsen GW, Bech C, Chastel O. 2010 Long-term survival effect of corticosterone manipulation in black-legged kittiwakes. Gen. Comp. Endocrinol. **167**, 246-251. (10.1016/j.ygcen.2010.03.018) 20338171

[18] Schultner J, Moe B, Chastel O, Tartu S, Bech C, Kitaysky A. 2014 Corticosterone mediates carry-over effects between breeding and migration in the kittiwake *Rissa tridactyla*. Mar. Ecol. Prog. Ser. **496**, 125-133. (10.3354/meps10603)

[19] Cockrem JF. 2013 Individual variation in glucocorticoid stress responses in animals. Gen. Comp. Endocrinol. **181**, 45-58. (10.1016/j.ygcen.2012.11.025) 23298571

[20] Andrews C et al. 2017 A marker of biological age explains individual variation in the strength of the adult stress response. R. Soc. Open Sci. **4**, 171208. (10.1098/rsos.171208) 28989794 PMC5627134

[21] Monaghan P. 2014 Organismal stress, telomeres and life histories. J. Exp. Biol. **217**, 57-66. (10.1242/jeb.090043) 24353204

[22] Reichert S, Stier A. 2017 Does oxidative stress shorten telomeres *in vivo*? A review. Biol. Lett. **13**, 20170463. (10.1098/rsbl.2017.0463) 29212750 PMC5746531

[23] Haussmann MF, Winkler DW, Huntington CE, Nisbet ICT, Vleck CM. 2007 Telomerase activity is maintained throughout the lifespan of long-lived birds. Exp. Gerontol. **42**, 610-618. (10.1016/j.exger.2007.03.004) 17470387

[24] Vedder O, Verhulst S, Bauch C, Bouwhuis S. 2017 Telomere attrition and growth: a life-history framework and case study in common terns. J. Evol. Biol. **30**, 1409-1419. (10.1111/jeb.13119) 28524249

[25] Tricola GM et al. 2018 The rate of telomere loss is related to maximum lifespan in birds. Phil. Trans. R. Soc. B **373**, 20160445. (10.1098/rstb.2016.0445) 29335369 PMC5784065

[26] Bauch C, Riechert J, Verhulst S, Becker PH. 2016 Telomere length reflects reproductive effort indicated by corticosterone levels in a long-lived seabird. Mol. Ecol. **25**, 5785-5794. (10.1111/mec.13874) 27696588

[27] Bauer CM, Graham JL, Abolins-Abols M, Heidinger BJ, Ketterson ED, Greives TJ. 2018 Chronological and biological age predict seasonal reproductive timing: an investigation of clutch initiation and telomeres in birds of known age. Am. Nat. **191**, 777-782. (10.1086/697224) 29750556

[28] Herborn K, Heidinger B, Boner W, Noguera JC, Adam A, Daunt F, Monaghan P. 2014 Stress exposure in early post-natal life reduces telomere length: an experimental demonstration in a long-lived seabird. Proc. R. Soc. B **281**, 20133151. (10.1098/rspb.2013.3151) PMC3973262 24648221

[29] Romero-Haro AÁ, Morger J, Haussmann MF, Tschirren B. In press. Reproductive strategies affect telomere dynamics across the life course. Am. Nat. 720440. (10.1086/720440) 35977791

[30] Angelier F, Weimerskirch H, Barbraud C, Chastel O. 2019 Is telomere length a molecular marker of individual quality? Insights from a long-lived bird. Funct. Ecol. **33**, 1076-1087. (10.1111/1365-2435.13307)

[31] Le Vaillant M, Viblanc VA, Saraux C, Le Bohec C, Le Maho Y, Kato A, Criscuolo F, Ropert-Coudert Y. 2015 Telomere length reflects individual quality in free-living adult king penguins. Polar Biol. **38**, 2059-2067. (10.1007/s00300-015-1766-0)

[32] Giraudeau M, Angelier F, Sepp T. 2019 Do telomeres influence pace-of-life-strategies in response to environmental conditions over a lifetime and between generations? Bioessays **41**, 1800162. (10.1002/bies.201800162) 30793350

[33] Sudyka J. 2019 Does reproduction shorten telomeres? Towards integrating individual quality with life-history strategies in telomere biology. Bioessays **41**, 1900095. (10.1002/bies.201900095) 31577044

[34] Angelier F, Costantini D, Blévin P, Chastel O. 2018 Do glucocorticoids mediate the link between environmental conditions and telomere dynamics in wild vertebrates? A review. Gen. Comp. Endocrinol. **256**, 99-111. (10.1016/j.ygcen.2017.07.007) 28705731

[35] Haussmann MF, Marchetto NM. 2010 Telomeres: linking stress and survival, ecology and evolution. Curr. Zool. **56**, 714-727. (10.1093/czoolo/56.6.714)

[36] Fokidis HB, des Roziers MB, Sparr R, Rogowski C, Sweazea K, Deviche P. 2012 Unpredictable food availability induces metabolic and hormonal changes independent of food intake in a sedentary songbird. J. Exp. Biol. **215**, 2920-2930. (10.1242/jeb.071043) 22837467

[37] Whelan S, Hatch SA, Benowitz-Fredericks ZM, Parenteau C, Chastel O, Elliott KH. 2021 The effects of food supply on reproductive hormones and timing of reproduction in an income-breeding seabird. Horm. Behav. **127**, 104874. (10.1016/j.yhbeh.2020.104874) 33191199

[38] Gill VA, Hatch SA. 2002 Components of productivity in black-legged kittiwakes *Rissa tridactyla*: response to supplemental feeding. J. Avian Biol. **33**, 113-126. (10.1034/j.1600-048X.2002.330201.x)

[39] Jacobsen K-O, Erikstad KE, Saether B-E. 1995 An experimental study of the costs of reproduction in the kittiwake *Rissa tridactyla*. Ecology **76**, 1636-1642. (10.2307/1938164)

[40] Tremblay F, Whelan S, Choy ES, Hatch SA, Elliott KH. 2022 Resting costs too: the relative importance of active and resting energy expenditure in a sub-arctic seabird. J. Exp. Biol. **225**, jeb.243548. (10.1242/jeb.243548) PMC8920031 35019973

[41] Romero LM, Reed JM. 2005 Collecting baseline corticosterone samples in the field: is under 3 min good enough? Comp. Biochem. Physiol. A. Mol. Integr. Physiol. **140**, 73-79. (10.1016/j.cbpb.2004.11.004) 15664315

[42] Welcker J, Speakman JR, Elliott KH, Hatch SA, Kitaysky AS. 2015 Resting and daily energy expenditures during reproduction are adjusted in opposite directions in free-living birds. Funct. Ecol. **29**, 250-258. (10.1111/1365-2435.12321)

[43] Osborne O, O'Hara P, Whelan S, Zandbergen P, Hatch S, Elliott K. 2020 Breeding seabirds increase foraging range in response to an extreme marine heatwave. Mar. Ecol. Prog. Ser. **646**, 161-173. (10.3354/meps13392)

[44] Young RC, Welcker J, Barger CP, Hatch SA, Merkling T, Kitaiskaia EV, Haussmann MF, Kitaysky AS. 2017 Effects of developmental conditions on growth, stress and telomeres in black-legged kittiwake chicks. Mol. Ecol. **26**, 3572-3584. (10.1111/mec.14121) 28370751

[45] Cawthon RM. 2002 Telomere measurement by quantitative PCR. Nucleic Acids Res. **30**, e47. (10.1093/nar/30.10.e47) 12000852 PMC115301

[46] Criscuolo F, Bize P, Nasir L, Metcalfe NB, Foote CG, Griffiths K, Gault EA, Monaghan P. 2009 Real-time quantitative PCR assay for measurement of avian telomeres. J. Avian Biol. **40**, 342-347. (10.1111/j.1600-048X.2008.04623.x)

[47] Foote C. 2008 Avian telomere dynamics. Doctoral dissertation, University of Glasgow, Glasgow, UK.

[48] Morinha F, Magalhães P, Blanco G. 2020 Standard guidelines for the publication of telomere qPCR results in evolutionary ecology. Mol. Ecol. Resour. **20**, 635-648. (10.1111/1755-0998.13152) 32133733

[49] Michelot T, Langrock R, Patterson TA. 2016 moveHMM: an R package for the statistical modelling of animal movement data using hidden Markov models. Methods Ecol. Evol. **7**, 1308-1315. (10.1111/2041-210X.12578)

[50] Patterson TA, Parton A, Langrock R, Blackwell PG, Thomas L, King R. 2017 Statistical modelling of individual animal movement: an overview of key methods and a discussion of practical challenges. AStA Adv. Stat. Anal. **101**, 399-438. (10.1007/s10182-017-0302-7)

[51] Vincenzi S, Hatch S, Mangel M, Kitaysky A. 2013 Food availability affects onset of reproduction in a long-lived seabird. Proc. R. Soc. B **280**, 20130554. (10.1098/rspb.2013.0554) PMC3652464 23576791

[52] van Noordwijk AJ, de Jong G. 1986 Acquisition and allocation of resources: their influence on variation in life history tactics. Am. Nat. **128**, 137-142. (10.1086/284547)

[53] Irons DB. 1998 Foraging area fidelity of individual seabirds in relation to tidal cycles and flock feeding. Ecology **79**, 10. (10.1890/0012-9658(1998)079[0647:FAFOIS]2.0.CO;2)

[54] Suryan R, Irons D, Kaufman M, Benson J, Jodice P, Roby D, Brown E. 2002 Short-term fluctuations in forage fish availability and the effect on prey selection and brood-rearing in the black-legged kittiwake *Rissa tridactyla*. Mar. Ecol. Prog. Ser. **236**, 273-287. (10.3354/meps236273)

[55] Müller C, Almasi B, Roulin A, Breuner CW, Jenni-Eiermann S, Jenni L. 2009 Effects of corticosterone pellets on baseline and stress-induced corticosterone and corticosteroid-binding-globulin. Gen. Comp. Endocrinol. **160**, 59-66. (10.1016/j.ygcen.2008.10.018) 18996387

[56] Torres-Medina F, Cabezas S, Marchant TA, Wikelski M, Romero LM, Hau M, Carrete M, Tella JL, Blas J. 2018 Corticosterone implants make stress hyporesponsive birds. J. Exp. Biol. **221**, jeb.173864. (10.1242/jeb.173864) 30111557

[57] Goutte A, Clement-Chastel C, Moe B, Bech C, Gabrielsen GW, Chastel O. 2011 Experimentally reduced corticosterone release promotes early breeding in black-legged kittiwakes. J. Exp. Biol. **214**, 2005-2013. (10.1242/jeb.051979) 21613516

[58] Bichet C, Bouwhuis S, Bauch C, Verhulst S, Becker PH, Vedder O. 2020 Telomere length is repeatable, shortens with age and reproductive success, and predicts remaining lifespan in a long-lived seabird. Mol. Ecol. **29**, 429-441. (10.1111/mec.15331) 31841253

[59] Hall ME, Nasir L, Daunt F, Gault EA, Croxall JP, Wanless S, Monaghan P. 2004 Telomere loss in relation to age and early environment in long-lived birds. Proc. R. Soc. Lond. B **271**, 1571-1576. (10.1098/rspb.2004.2768) PMC1691772 15306302

[60] Watson H, Bolton M, Monaghan P. 2015 Variation in early-life telomere dynamics in a long-lived bird: links to environmental conditions and survival. J. Exp. Biol. **218**, 668-674. (10.1242/jeb.104265) 25617465 PMC4376192

[61] Zane L, Ensminger DC, Vázquez-Medina JP. 2021 Short-term elevations in glucocorticoids do not alter telomere lengths: a systematic review and meta-analysis of non-primate vertebrate studies. PLoS ONE **16**, e0257370. (10.1371/journal.pone.0257370) 34597314 PMC8486123

[62] Gillies N et al. 2020 Short-term behavioral impact contrasts with long-term fitness consequences of biologging in a long-lived seabird. Sci. Rep. **10**, 15056. (10.1038/s41598-020-72199-w) 32929167 PMC7490266

[63] Bateson M, Nettle D. 2018 Why are there associations between telomere length and behaviour? Phil. Trans. R. Soc. B **373**, 20160438. (10.1098/rstb.2016.0438) 29335363 PMC5784059

[64] Bateson M, Brilot BO, Gillespie R, Monaghan P, Nettle D. 2015 Developmental telomere attrition predicts impulsive decision-making in adult starlings. Proc. R. Soc. B **282**, 20142140. (10.1098/rspb.2014.2140) PMC4286045 25473012

[65] Satterthwaite WH, Kitaysky AS, Hatch SA, Piatt JF, Mangel M. 2010 Unifying quantitative life-history theory and field endocrinology to assess prudent parenthood in a long-lived seabird. Evol. Ecol. Res. **12**, 14.

[66] Benowitz-Fredericks ZM, Lacey LM, Whelan S, Will AP, Hatch SA, Kitaysky AS. 2022 Data from: Telomere length correlates with physiological and behavioural responses of a long-lived seabird to an ecologically relevant challenge. Dryad Digital Repository. (10.5061/dryad.v9s4mw6xs) PMC9277278 35858061

[67] Benowitz-Fredericks ZM, Lacey LM, Whelan S, Will AP, Hatch SA, Kitaysky AS. 2022 Telomere length correlates with physiological and behavioural responses of a long-lived seabird to an ecologically-relevant challenge. *Figshare*. (10.6084/m9.figshare.c.6060559) PMC9277278 35858061

